# Successful medical treatment of fetal supraventricular tachycardia that cause hydrops fetalis

**DOI:** 10.4274/tjod.56578

**Published:** 2014-09-15

**Authors:** Cihan Çetin, Çiğdem Akçabay, Selim Büyükkurt, Nazan Özbarlas

**Affiliations:** 1 Çukurova University Faculty of Medicine, Department of Obstetrics and Gynecology, Adana, Turkey; 2 Elazığ Research and Education Hospital, Clinic of Obstetrics and Gynecology, Elazığ, Turkey; 3 Çukurova University Faculty of Medicine, Department of Pediatric Cardiology, Adana, Turkey

**Keywords:** Tachycardia, supraventricular, fetal hydrops

## Abstract

Supraventricular tachycardia (SVT) is the most frequent fetal tachyarrhythmia. Diagnosis is established with M-mode ultrasound and/or Doppler investigation. Untreated cases may develop fetal heart failure and hydrops. Even these cases should not be left untreated - maternal administration of anti-arrhythmic drugs should be undertaken. In this manuscript, we describe a successful treatment with maternal administration of sotalol and digoxin in a fetus that developed hydrops because of SVT.

## INTRODUCTION

Supraventricular tachycardia (SVT) is the most frequent fetal tachyarrhythmia^(1)^. There was difficulty in the prenatal diagnosis of fetal arrhythmias before the advances in the ultrasound technology. Currently, they can be identified in detail. Especially, M-mode echocardiography is very helpful in the identification of fetal arrhythmias. SVT, if untreated or overlooked by the inadequate evaluation, may cause ventricular dysfunction and hydops fetalis. The most important factor in progression to hydrops fetalis is the heart rate and duration and progression rate of SVT^(2)^. Untreated cases of hydrops may easily cause death of fetus or hypoxic events caused by the impaired systemic perfusion in the fetus. In the cases of fetal SVT’s, there are various management strategies at different clinics depending on their own experiences. These include expectant management, birth and postnatal treatment, fetal intramuscular injections and maternal administration of drugs^(3)^. Drugs used in the prenatal period include digoxin, sotalol, flecainide and amiodarone^(4)^. In this manuscript, we present the treatment of a hydropic fetus by the maternal administration of digoxin and sotalol.

## CASE

**Figure 1:**
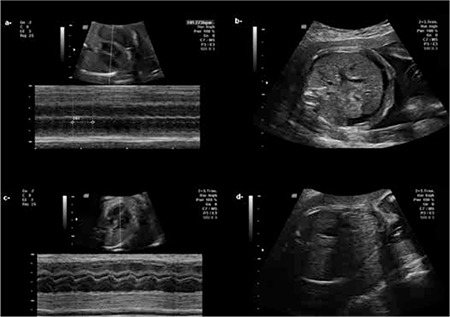
Before the treatment, M-mode shows tachycardia in which every atrial contraction follows a ventricular contraction (a) and the presence of ascites (b). At the 11^th^ day of treatment, heart rate returned to sinus rhythm (c) and ascites completely resolved (d)

Patient was referred to our prenatal diagnosis center during her 24 weeks of gestation due to fetal ascites. Upon evaluation, the patient did not have any prior significant medical or surgical history. Ultrasonographic evaluation revealed fetal ascites with fetal supraventricular tachycardia. No additional finding that may cause ascites was found during ultrasound examination. Fetal heart rate was 273 beats/min and every atrial contraction was followed by a ventricular contraction. Patient was given digoxin (bolus: 500 μg 2x1 PO, maintenance: 250 μg 2x1 PO) and sotalol (160 mg 2x1 PO) for four days. Patient was followed by electrocardiography (ECG) (especially corrected QT interval) and blood digoxin levels. Due to persistant fetal tachycardia, sotalol dose was elevated to 320 mg 3x1 in the following four days. On the eigth day of treatment fetaus was on sinus rhythm and heart rate was 140 beats/min. During the follow-up period, no tachycardia episodes were identified and on account of this sotalol dose gradually decreased to 80 mg 2x1 on the 13^th^ day of treatment. Fetal ascites completely resolved on the 11^th^ day of treatment (Figure 1). On the third week of treatment sotalol dose remained same, whereas digoxin dose was decreased to 250 μg 1x1. During the rest of the pregnancy no episodes of fetal tachycardia and ascites was observed at ultrasonographic evaluations. Patient delivered a male fetus vaginally on the 38 weeks of gestation and infant had no complication (cardiac, neurologic etc.) during the postnatal six months period.

## DISCUSSION

Hydrops fetalis is usually divided into two categories: İmmune and non-immune hydrops fetalis. The most important etiologic factor for immune hydrops fetalis is Rh isoimmunization and this is markedly decreased in the past three- four decades by the effective administration of prophylactic regimens; currently most of the hydops fetalis cases were in the category of non-immune group and the etiology of these are multifactorial^(5)^. Despite the advances in the etiopatogenesis and treatment of hydrops fetalis, mortality rate still remains near 50%^(5,6)^. As it can be thought, survival without neurologic sequela is even less.

Especially, M-mode should be chosen during the evaluation of arrhythmias with the ultrasound. M-mode line should be placed passing from both atrium and ventricle so that evaluation of the relation between atrial and ventricular contractions can be made. Another option is the placement of the line passing from both atrium and aorta. By this way, atrial rate, ventricular rate, relation between atrial and ventricular beats and the type of the arrhythmia can be determined.

Sotalol is a beta adrenergic reseptor blocker class III 3 antiarrhythmic drug that prolongs the cardiac repolarization interval^(7)^. Like other antiarrhythmic drugs, sotalol has also proarrhythmic potential. It is especially chosen in the treatment of fetal arrhythmias due to its fast and adequate crossing from the placenta^(7)^. Digoxin exhibits its effect by both increasing the refractoriness at the atrioventricular node and having positive inotrophic effect. Digoxin should not be used alone in the treatment of fetal SVT due to the potential existence of an atrioventricular accessory pathway^(8)^. It is usually used together with drugs like sotalol or flecainide. Digoxin is also thought to be effective in the hydropic cases due to its positive inotrophic effect^(8)^.

One of the other drugs used in the antiarrhythmic treatments, flecainide needs serum level follow-up and another drug, amiodarone has potential risk of developing fetal hypothyroidism; on account of these, sotalol is thought to be safer in the first-line treatment^(9,10,11,12)^. Only ECG follow-up at certain periods is enough for the patients given sotalol^(13)^.

The recommended bolus dose is between 250-1500 μg and maintanence dose is between 125-500 μg for digoxin^(3)^. Although the recommended dose for sotalol is 160 mg PO two times a day, it can be increased to maximum dose of 160mg PO three times a day. Whereas we achieved to treat SVT in our patient by 320 mg three times a day. However, we did not encounter any maternal side effect.

The major concern with the use of maternally administered drugs is their proarrythmic effect (especially long QT on ECG). QT interval may be effected by the heart rate, on). Multiple studies in the literature suggest not to avoid the use of these drugs especially in the pregnant patients, because of their very low potential for such an adverse effect^(9,13)^. After the resolution of fetal tachycardia, the patient should be followed with the minimum maintenance dose. Another tool in the following of digoxin theraphy is the blood level of the drug. When blood digoxin levels exceeds 1.3-1.5 ng/mL, signs of toxicity may appear. In order to determine the correct blood level of digoxin, blood sample should be taken at least six hours after the intake of last dose. By the resolution of tachycardia, fetal monitoring during the labor becomes easier and unnecessary ceserean rates would decrease, also.

Moodle et al. defined hydrops fetalis, female fetus and unsuccessful resolution of the arrythmia as potential risk factors for postnatal arrythmia^(14)^. As compatible with this, male fetus and successful return to sinus rhythm prenatally was thought good prognostic factors in our case; however, despite the existence of poor prognostic factor hydrops fetalis, no postnatal arrythmia was observed.

In conclusion, the aim should be to treat fetal tachycardia before the development of hydrops. However, even for the cases in which hydrops already developed, treatment should not be avoided, and optimal drug dose for mother and the fetus should be given. The following of medical treatment should be done by both maternal and fetal parameters. Although no standart dosing of the drugs has been established yet, our case is one of the few cases describing the treatment experience in this condition in our country.

## DISCUSSION

Sirenomelia, a developmental defect that involves the caudal region of the body, is associated with several internal visceral anomalies. It is associated with renal agenesis, sacral agenesis, anorectal atresia, imperforate anus, absent urinary bladder, lumbosacral and pelvic bone abnormalities, single umbilical artery, and ambiguous genitalia. Sirenomelia is fatal in most cases because of the characteristic pulmonary hypoplasia due to the severe oligohydramnios^(4)^. Two theories have been proposed to explain the etiopathogenesis of sirenomelia; the vascular steal hypothesis and the defective blastogenesis hypothesis. Normally, the umbilical cord contains two arteries that originate from the iliac arteries, which return blood to the placenta. In cases of sirenomelia, the umbilical artery is single and arises from the abdominal aorta. The abdominal aorta distal to this branch directly bifurcates into iliac iliac arteries without giving an origin to renal or inferior mesenteric artery branches. These vascular abnormalities lead to vitelline artery steal of the blood supply to the caudal end of embryo, which leads to sirenomelia and associated anomalies^(5)^. At blastogenesis, damage to the caudal mesoderm of the embryo between day 13 and day 22 of life results in merging, malrotation, and dysgenesis of the lower extremities.

A diagnosis of sirenomelia may be easier to make during the first trimester because the amniotic fluid volume is relatively normal, because amniotic fluid is secreted by the amniotic membrane in the first trimester^(6)^. A diagnosis of sirenomelia is made in early pregnancy through confirmation of the existence of a single lower extremity. At later periods of pregnancy, ultrasonographic diagnosis of sirenomelia may be prevented by severe oligohydramnios due to renal agenesis or dysgenesis. Our patient was referred to us late in the first trimester.
